# Ectopic Recombination of a Malaria *var* Gene during Mitosis Associated with an Altered *var* Switch Rate

**DOI:** 10.1016/j.jmb.2009.04.032

**Published:** 2009-06-12

**Authors:** Michael F. Duffy, Timothy J. Byrne, Celine Carret, Alasdair Ivens, Graham V. Brown

**Affiliations:** 1 Department of Medicine at RMH, University of Melbourne, Parkville 3050, Australia; 2 Pathogen Microarrays Group, The Wellcome Trust Sanger Institute, Hinxton, Cambridge CB10 1SA, UK

**Keywords:** PfEMP1, *P. falciparum* erythrocyte membrane protein 1, PFGE, pulsed-field gel electrophoresis, TARE, telomere-associated repetitive element, Q-RT-PCR, quantitative reverse transcription–polymerase chain reaction, ICAM-1, intercellular adhesion molecule 1, CI, confidence interval, malaria, recombination, *var*, switch, transcription

## Abstract

The *Plasmodium falciparum var* multigene family encodes *P. falciparum* erythrocyte membrane protein 1, which is responsible for the pathogenic traits of antigenic variation and adhesion of infected erythrocytes to host receptors during malaria infection. Clonal antigenic variation of *P. falciparum* erythrocyte membrane protein 1 is controlled by the switching between exclusively transcribed *var* genes. The tremendous diversity of the *var* gene repertoire both within and between parasite strains is critical for the parasite's strategy of immune evasion. We show that ectopic recombination between *var* genes occurs during mitosis, providing *P. falciparum* with opportunities to diversify its *var* repertoire, even during the course of a single infection. We show that the regulation of the recombined *var* gene has been disrupted, resulting in its persistent activation although the regulation of most other *var* genes is unaffected. The *var* promoter and intron of the recombined *var* gene are not responsible for its atypically persistent activity, and we conclude that altered subtelomeric cis sequence is the most likely cause of the persistent activity of the recombined *var* gene.

## Introduction

Each *Plasmodium falciparum* parasite possesses approximately 60 members of the *var* multigene family, which encode variants of *P. falciparum* erythrocyte membrane protein 1 (PfEMP1), the immunodominant variant antigen of *P. falciparum.* PfEMP1 is expressed on the surface of the infected erythrocyte and is the parasite ligand that binds numerous host receptors in vascular tissues, thus mediating the pathogenic traits of infected erythrocyte sequestration in these tissues and rosetting. *Var* genes show tremendous diversity among parasites,^1^ and it has been proposed that much of this diversity arises from ectopic recombination between regions of homology within *var* genes leading to a vast repertoire of chimaeric *var* gene sequences.^2^ This has been demonstrated for meiosis and has been proposed to occur during mitosis.^3^

A single parasite abundantly transcribes a single *var* gene at one time and *P. falciparum* evades the host immune response through antigenic variation by switching between the dominant transcribed *var* genes. Partially defined epigenetic processes control the transcription of a single *var* gene. Histone deacetylation has been shown to silence some *var* genes,^4^ and specific histone methylation and acetylation modifications are associated with *var* gene silencing and activation.^5,6^

*Var* genes are present as clusters or single copies at chromosome internal or subtelomeric sites with upstream sequences (ups) defined by homology as being upsA, B, C, or E.^2,7^ The different ups types have a restricted genomic distribution: upsC are present in chromosome internal *var* genes; upsB are present in the most subtelomeric *var* genes, which are transcribed towards the centromere and in some chromosome internal *var* genes; upsA are present in *var* genes that are transcribed towards the telomere and that are also at subtelomeric sites but are centromeric to upsB *var* genes; upsE, which are always upstream of the conserved *var2csa* gene, have the same chromosomal location and orientation as the upsA *var* genes.^2^ The different orientations of the different ups type *var* genes have been proposed to restrict their ability to recombine to other *var* genes of the same type, thus forming compartments of *var* genes within the genomic repertoire.^2^ The rate at which *var* genes switch on and off is a fixed, gene-specific characteristic.^8^ The on and off rates are different so that genes that switch on rapidly and off slowly should predominate in a parasite in the absence of any selective pressure on PfEMP1. A recent study revealed that different ups types have different on- and off-switch rates with upsC *var* genes switching off much more slowly than upsA, upsB, or upsE.^9^ Whether the switch rate was dependent on the ups type, on the chromosomal location of the *var* gene, or on some other cis sequence element associated with the *var* gene locus could not be determined. Some cis sequence elements have been associated with *var* gene silencing including upstream elements^10^ and the *var* gene intron that possesses both bidirectional promoter activity^11^ and the ability to repress the activity of *var* gene promoters.^12^

We report the first evidence of a parasite that has undergone ectopic recombination within a *var* gene during mitosis to generate progeny carrying a chimaeric *var* gene. This recombination has occurred within the *var* gene intron and has generated a functional chimaeric *var* gene that has altered activity in that it is more rapidly turned on and persistently transcribed. The ability to switch the expression of *var* genes on and off at other loci appears unaffected, suggesting that the effect on *var* gene switching is due to the recombination. The chimaeric *var* gene remains in its original locus and its promoter is unaffected. We showed that subtelomeric repeat sequence could decrease *var* promoter activity and conclude that *var* gene regulation is not determined solely by upstream sequence or genomic location.

## Results

### Ectopic recombination within a *var* gene during mitosis

**Fig. 1:**
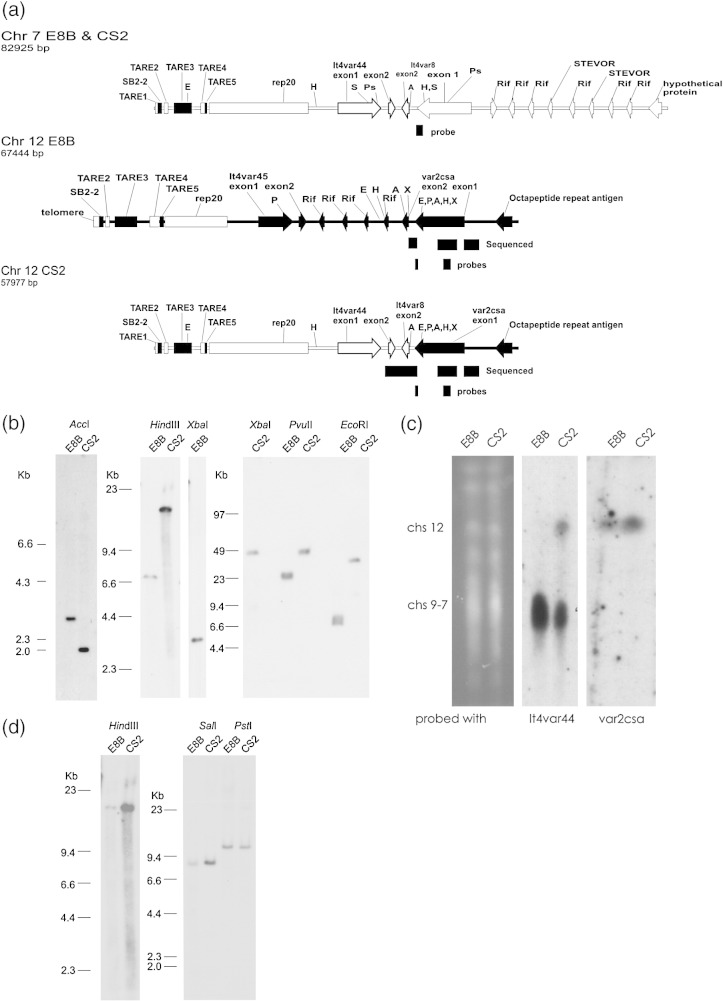
(a) Diagrams of the left-hand telomeric fragment of chromosome 7 and the *var2csa* locus on the left-hand telomeric fragment of chromosome 12 in wild-type E8B clonal parasites and in their clonal progeny CS2 following ectopic recombination between chromosome 7 and chromosome 12. The filled bar indicates wild-type chromosome 12, and the unfilled bar indicates the sequence from chromosome 7 introduced following recombination. Positions are indicated for TARE 1–5, TARE 6 (rep20), the second telomere-associated repeat sequence from subtelomeric block 2 (SB2-2),^7^ several members of the rif and *var* multigene families, and the restriction sites AccI (A), EcoRI (E), HindIII (H), PvuII (P), XbaI (X), SalI (S), and PstI (Ps). The positions of probes used for Southern and Northern blots and regions that were directly sequenced are indicated beneath the diagrams. (b) Southern blots of CS2 and E8B gDNA digested with the enzymes indicated above the figure and separated by conventional gels (AccI, HindIII, and XbaI) and PFGE (XbaI, PvuII, and EcoRI) were probed with sequence from the 3′ end of *var2csa* exon 1. (c) Southern blots of PFGE-separated chromosomes probed with sequence from the 3′ ends of *var2csa* and It4_var44. (d) Southern blots of CS2 and E8B gDNA digested with the enzymes indicated above the figure were probed with sequence from the 3′ end of It4_var8 exon 1.

Ectopic recombination during mitotic division of the CS2 clonal parasite has replaced the *var2csa* intron, the second exon, and sequence downstream of *var2csa* with sequences originating within the *var* gene It4_var8 (Fig. 1a). The CS2 parasite is a mitotic progeny of the parental E8B clone and was derived from E8B by selection for adhesion to chondroitin sulfate A. The E8B parasite was itself cloned from ItG parasites. The CS2 parasites described in this study were cloned again prior to analysis. PCR using a degenerate second exon primer was used to obtain CS2 *var2csa* intron and second exon sequence. Universal Fast Walking PCR^13^ was used to obtain 2645 nucleotides of unknown sequence downstream of the *var2csa* second exon (Fig. 1a). Recombination within the *var2csa* intron was confirmed by Southern blotting AccI-digested DNA (Fig. 1b) and sequencing cloned PCR products that were amplified using specific primers on gDNA. Pulsed-field gel electrophoresis (PFGE) was used to confirm retention of *var2csa* on chromosome 12 in CS2 parasites and to identify the original location of the sequence integrated into the *var2csa* locus as either chromosome 7 or chromosome 8 (Fig. 1c). PFGE revealed that the genetic rearrangement was nonreciprocal, with the original sequence also remaining in the original locus on chromosome 7or 8 in CS2.

We used ItG sequence data produced by the *P. falciparum* Sequencing Group at the Sanger Institute that can be obtained from ftp://ftp.sanger.ac.uk/pub/pathogens/Plasmodium/falciparum/IT_strain/ to identify sequences contiguous with those we had cloned. The ItG Supercontig_0002004 starts at telomere-associated repetitive element (TARE) 1, which is usually adjacent to the approximately 1.5-kb-long *P. falciparum* telomere sequence,^14^ and continues through TARE 1–6,^15^ It4_var44, and It4_var8. There is 41 kb between the start of Supercontig_0002004 and the recombination site within the It4_var8 intron. The Supercontig_0002007 available at ftp://ftp.sanger.ac.uk/pub/pathogens/Plasmodium/falciparum/IT_strain/ contains sequence including the 50 kb between the left-hand end of chromosome 12 and the recombination site in the *var2csa* intron. From this resource, a single copy conserved gene contiguous with It4_var8 was identified on the Sanger supercontig_0002004 that is also present as PF07_0004 at a subtelomeric site on chromosome 7 in 3D7 parasites. We concluded that the sequence integrated into *var2csa* came from chromosome 7.

**Supplementary Fig. 1:**
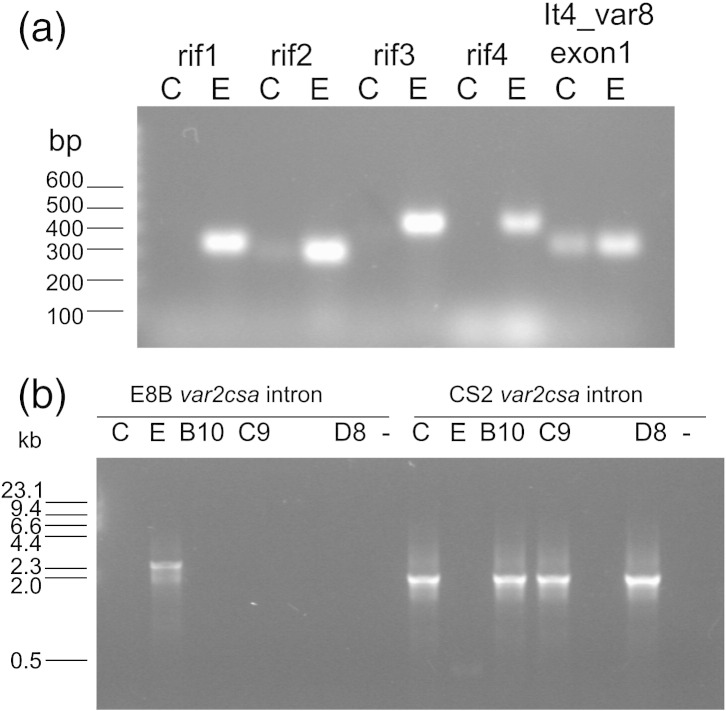
(a) PCR of the four rif genes present on chromosome 12 of E8B (E) and lost following recombination in CS2 (C); a PCR of It4_var8 exon 1 was included as a positive control. (b) PCR across the *var2csa* intron using primers specific for the E8B wild-type sequence or the CS2 mutant sequence: CS2 (C), E8B (E), CS2 ICAM-1-selected subclones CS2isB10 (B10), CS2isC9 (C9), CS2isD8 (D8), negative control (-). Subclone C8 could not be recovered from cryopreserves for this analysis.

The Southern blot hybridisation patterns of CS2 and E8B gDNA digested with EcoRI, HindIII, PvuII, and XbaI and probed with sequence from the 3′ end of *var2csa* exon 1 all confirmed that a duplicative transposition had occurred (Fig. 1b). An 18-kb HindIII fragment and a 36.8-kb EcoRI fragment from CS2 parasites hybridised to a probe from the 3′ end of *var2csa* exon 1 and confirmed that the left-hand end of chromosome 12 from the *var2csa* intron to at least the subtelomeric repeats had been replaced with chromosome 7 donor sequence that extended from near the start of the It4_var8 intron through the HindIII site between the It4var44 exon 1 and rep20 repeat sequences and to at least the EcoRI site in TARE 3. Replacement of the wild-type ItG chromosome 12 sequence with chromosome 7 resulted in loss of the PvuII and XbaI sites that generated the predicted fragments of 22 and 3.1 kb, respectively, from chromosome 12 in wild-type E8B parasites. The observed PvuII and XbaI fragments from chromosome 12 in CS2 parasites that hybridised to *var2csa* exon 1 were consistent with the predicted 43.6 XbaI and 42.5 PvuII fragments that would span from *var2csa* to the telomere if recombination with Supercontig_0002004 chromosome 7 sequence had resulted in replacement of the entire left-hand subtelomeric sequence of chromosome 12 with that of chromosome 7. PCR confirmed the loss from CS2 of the *rif* genes located between It4var_45 and *var2csa* on chromosome 12 of E8B (Supplementary Fig. 1)

Sequence from the first exon of It4_var8 hybridised on Southern blots to 11.35-kb PstI, 8.9-kb SalI, and 17.1-kb HindIII fragments of both CS2 and E8B gDNA, proving that the left-hand telomeric chromosome 7 sequence remained intact in CS2 from the PstI site halfway through the first exon of It4_var8 to at least the HindIII site between the It4var44 exon 1 and rep20 repeat sequences (Fig. 1d).

The recombined *var2csa* intron had a similar overall structure to other *var* introns (Supplementary Fig. 2). The three regions previously defined by strand asymmetry were present as were the repeats in the G-rich region 1 that were complementary to repeats present in the C-rich region 3.^11^ The number of these repeats was the same between CS2 *var2csa*, E8B *var2csa*, and It4_var8, although the spacing between the repeats was different. The intron region 2 that possesses bidirectional promoter activity was 526 nucleotides long in E8B *var2csa* and 449 nucleotides long in CS2 *var2csa*.

### The chimaeric *var2csa* gene is persistently transcribed

**Fig. 2:**
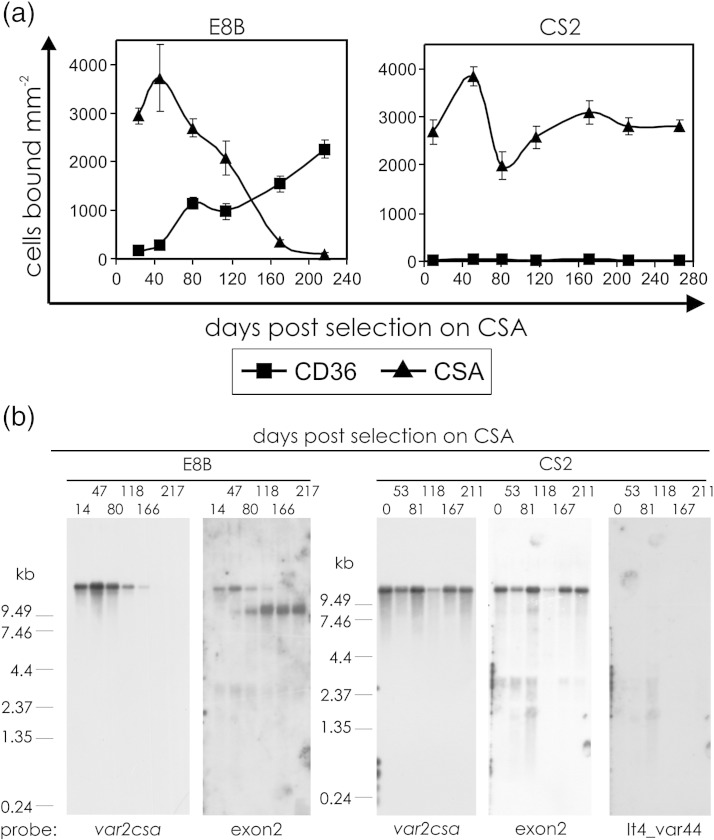
(a) The numbers of CS2 and E8B parasites that bound per square millimeter to immobilised CSA and CD36 at different time points following selection on CSA. Means of three replicates for each of two assays conducted on separate days; error bars are standard error of the mean. (b) Northern blots of RNA from E8B and CS2 parasites collected at different time points following selection on CSA and probed with sequence from the centre of the first exon of *var2csa* (Fig. 1a), from the 3′ end of IT4_var44, and with a conserved *var* exon 2 probe.

CS2 and E8B clonal parasites were selected for adhesion to CSA and grown for 266 and 217 days, respectively, in the absence of selection. Their adhesion phenotype and *var* gene transcriptional profile were tested at regular intervals over this time. CS2 parasites did not lose the ability to adhere to CSA over this period; however, the E8B parasites gradually lost the ability to adhere to CSA and reverted to the default CD36 adhesion phenotype (Fig. 2a). This reversion to CD36 adhesion by E8B parasites was accompanied by a decrease in transcription of *var2csa* and an increase in transcription of other *var* genes whilst CS2 parasites maintained their transcription of *var2csa* over the entire period (Fig. 2b).

### Persistent activity of *var2csa* is not associated with promoter mutation

To determine whether mutation or recombination of the upsE sequence upstream of the *var2csa* in CS2 may have caused the persistent *var2csa* activity, we amplified, cloned, and sequenced 2381 bp of upstream sequence in both CS2 and E8B parasites. The sequences were amplified on two separate occasions using different methods, and 5 clones for each upsE were fully sequenced. Over the 2381 bp, there was an average of 2.5 mismatches and one insertion or deletion per clone relative to the consensus derived from all 10 clones. In all but two instances, the mutations were present in only a single clone; in the two exceptions, there were single and dinucleotide insertions in homopolymeric tracts that were present in only 2 of the 5 clones available for that upsE sequence. Thus, we concluded that the E8B and CS2 upsE sequences were identical over the 2381 bp and that persistent *var2csa* transcription in CS2 was not due to differences in the upsE sequence.

### Persistent transcription of *var2csa* in CS2 parasites is not due to a general deficit in the *var* gene switching machinery

**Fig. 3:**
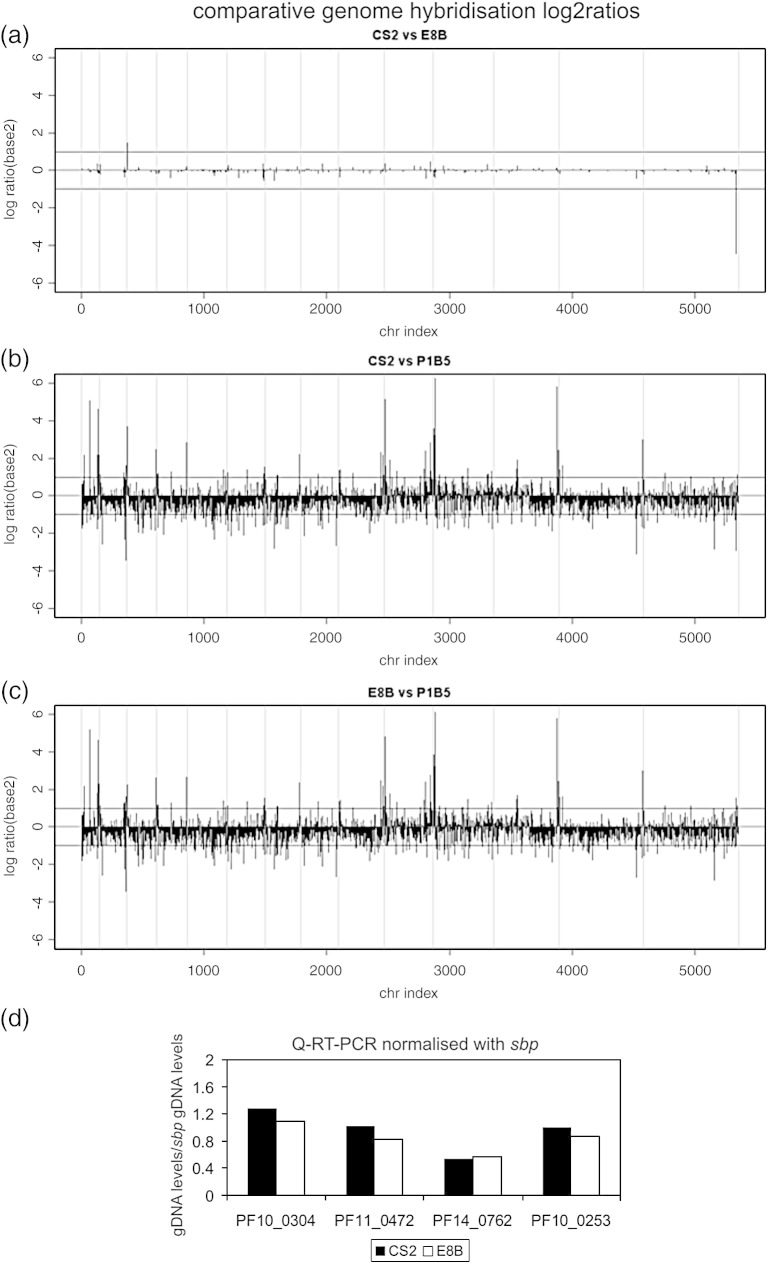
Comparative genome hybridisation log2ratios of (a) CS2 compared to E8B, (b) CS2 compared to P1B5, and (c) E8B compared to P1B5. The genes (vertical black bars) are plotted as physical order on the *x*-axis whilst the log ratios are on the *y*-axis. The zero line shows the distribution of the genes if no variation was to be found between the two strains compared. The horizontal lines at + 1 and − 1 are limits indicating amplification (log2ratio > 1) and deletion (log2ratio < − 1), respectively. The grey vertical bars delimit the chromosomes. (d) The skeleton binding protein gene *sbp* and genes identified as possibly deleted or duplicated by microarray were quantitated in both CS2 and E8B gDNA using standard curves of serially diluted gDNA. The levels of *sbp* were used to normalise the levels of the other genes so that the resulting relative values compare the level of each gene in equivalent amounts of CS2 and E8B gDNA.

To determine whether the persistent *var2csa* transcription was caused by a loss of genes controlling epigenetic regulation, we compared genomic DNA from CS2 and E8B parasites by microarray hybridisation. The 3D7 array used would not detect loss of genes specific to ItG lineage parasites, but we assumed that genes controlling the *var* gene switching mechanism would be conserved among *P. falciparum* isolates. There was very little genetic variation between E8B and CS2, confirming their mitotic, clonal lineage (Fig. 3). This finding contrasted with the polymorphism previously observed between Ituxi (IT) subclones including the sequenced clone P1B5. Comparative analysis with P1B5 revealed much more polymorphism between it and either CS2 or E8B than between CS2 and E8B, suggesting that the previously observed polymorphisms between IT subclones related to the source from which they were derived.^16^

Five genes had log2 CS2/E8B ratios ≥ 1 or ≤ − 1, indicating duplication or deletion (Supplementary Table 1). PFC0015c log2ratio CS2/E8B = 1.452014 is a subtelomeric VARC pseudogene, that is, *var* exon 2 sequence, which is unlikely to be conserved between 3D7 and ItG. The probeset for PFC0015c was limited to two oligonucleotides (PF03.4611c_st and PF03.4611w_st) that were, in fact, complementary; thus, the probeset only covered a single 25-nucleotide sequence that was 80% identical with exon 2 of It4_var8, 84% identical with exon 2 of It4_var44, and 88% identical with exon 2 of It4_var45 but had no identity to the wild-type E8B *var2csa* exon 2. Therefore, the recombination at the *var2csa* locus in CS2 led to a net gain of one sequence that could hybridise to these probes and PFC0015c was not investigated further.

The other genes that varied between CS2 and E8B were PF14_0762, a predicted exported protein of unknown function with a log2ratio(CS2/E8B) = − 4.45878, and three predicted proteins of unknown function, PF10_0304, PF11_0472, and PF10_0253, which had log2ratio(CS2/E8B) values of 1.032, 1, and 0.984, respectively. Quantitative reverse transcription–polymerase chain reaction (Q-RT-PCR) primers that amplified a region covered by the microarray probes were designed and used to quantitate the levels of these genes in CS2 and E8B gDNA (Fig. 3d). All of the genes were shown to be present at equivalent levels in CS2 and E8B gDNA; hence, we concluded that microarray analysis had not detected any amplifications or deletions in CS2 *versus* E8B apart from the net gain of one *var* exon 2 sequence with homology to the 3D7 VARC PFC0015c. Although this analysis was a useful screen for candidate regulators of switching, it could not detect recombinations or point mutations within genes encoding regulators of *var* gene switching.

**Fig. 4:**
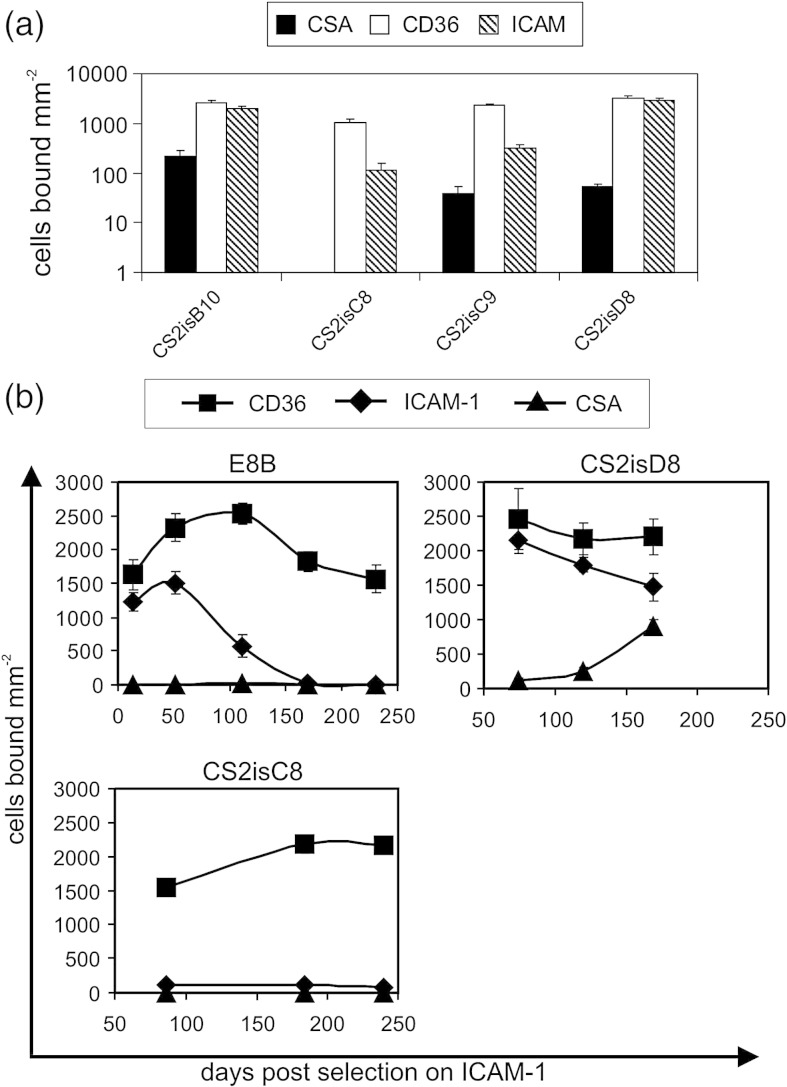
(a) CS2 parasites were selected on ICAM-1 and then cloned, and the number of infected erythrocytes of each clone that bound per square millimeter to immobilised CSA, CD36, and ICAM-1 was determined. (b) The numbers of erythrocytes infected with CS2 clones and E8B parasites that bound per square millimeter to immobilised CSA, CD36, and ICAM-1 at different time points following selection on ICAM-1. Means of three replicates for each of two assays conducted on separate days; error bars are standard error of the mean.

**Fig. 5:**
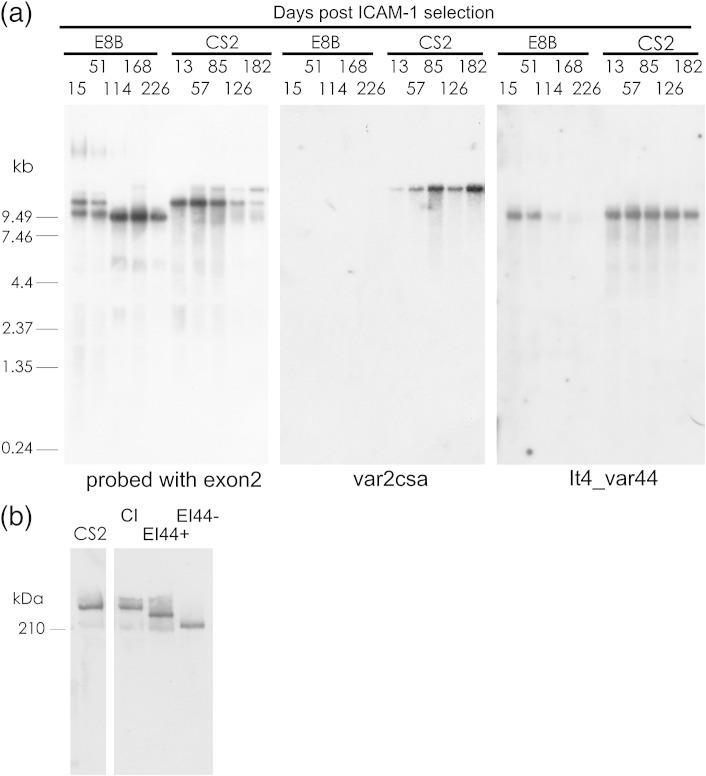
(a) Northern blot of RNA collected from E8B and CS2 parasites at different times after selection for adhesion to ICAM-1 and sequentially probed with sequences from *var2csa*, the 3′ end of exon 1 of It4_var44, and the conserved *var* exon 2. (b) Western blot probed with rabbit antiserum to the conserved exon 2 of PfEMP1; samples were CS2, CS2 selected on ICAM-1 and cultured until both *var2csa* and It4_var44 were transcribed (CI), E8B soon after selection on ICAM-1 when It4_var44 was transcribed (EI44+), and E8B selected on ICAM-1 and grown until transcription of It4_var44 had ceased (EI44-).

**Fig. 6:**
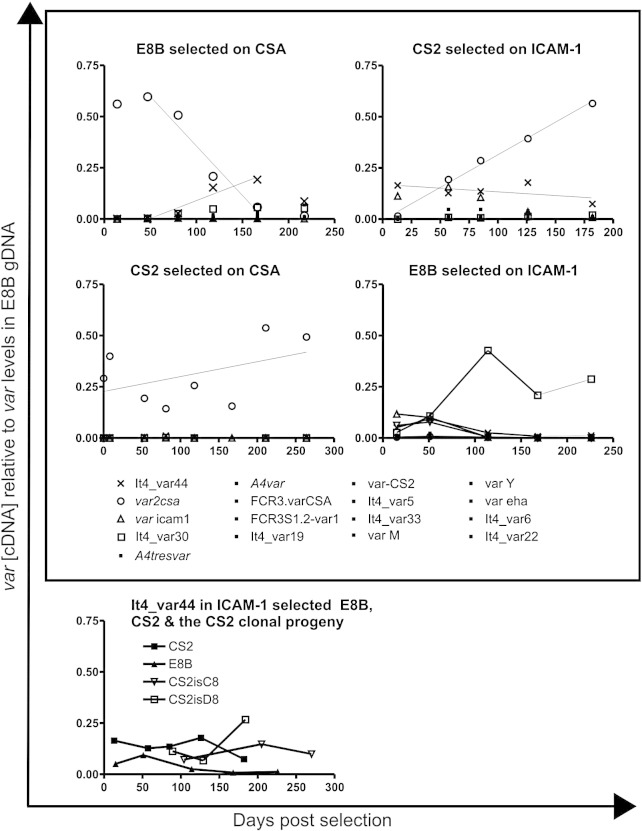
Q-RT-PCR was used to determine the levels of 17 *var* genes in the cDNAs of E8B and CS2 parasites at different time points during continuous growth following selection for adhesion to CSA and ICAM-1. Quantitation was performed using efficiency correction for each PCR and expressed relative to the levels of each gene in E8B gDNA following normalisation with the skeleton binding protein gene *sbp*. Linear regression curves are indicated on the plots of E8B selected on CSA and CS2 selected on CSA and ICAM-1.

To confirm that loss of trans factors involved in general epigenetic regulation was not responsible for the persistent transcription of *var2csa* in CS2, we analysed the ability of CS2 and E8B parasites to switch from expression of *var* genes encoding an intercellular adhesion molecule 1 (ICAM-1) adhesion phenotype. CS2 and E8B parasites were selected for adhesion to ICAM-1 and then the CS2 parasites were cloned prior to further analysis. This excluded the possibility that a minor population of CSA binding CS2 parasites persisted through the selection for ICAM-1 and then outgrew the ICAM-1 binding CS2 parasites. The four CS2 clones analysed bound predominantly to ICAM-1 and CD36, which can be encoded by the same *var* gene.^17^ However, three clones (CS2isB10, CS2isC9, and CS2isD8) also bound at low levels to CSA (Fig. 4a) and had therefore switched on *var2csa* expression during the 65 days between ICAM-1 selection and recovery of sufficient culture of the CS2 clones to perform adhesion assays. The ICAM-1-selected E8B and two of the ICAM-1-selected CS2 clones were then grown continuously *in vitro*. Both the ICAM-1-selected E8B and the CS2 clone CS2isD8 progressively lost adhesion to ICAM-1; the CS2 clone CS2isC8 had presumably already lost ICAM-1 adhesion during the period of cloning and subsequent growth (Fig. 4b). Both E8B and CS2 switched to transcription of other *var* genes (Figs. 5a and 6), proving that the persistent *var2csa* transcription was not due to a general defect in the ability to switch between transcription of different *var* genes due to the loss of factors regulating *var* gene switching. The ICAM-1-selected CS2 parasites spontaneously switched to transcription of *var2csa* when grown in the absence of phenotype selection but the parental E8B parasites selected on ICAM-1 did not (Figs. 5a and 6), demonstrating that *var2csa* had a higher on switch in CS2 than in the wild-type E8B.

### The *var2csa* on-switch rate is different between CS2 and E8B parasites

The off-switch rates for *var* genes in 3D7 were determined previously as approximately 1% for *var2csa* and between 0% and 0.3% for the upsC *var* genes.^9^ We estimated switch rates for *var2csa* using Q-RT-PCR. The cDNA levels of 17 ItG *var* genes were determined at different time points over several months of continuous culture following selection of CS2 and E8B parasites for adhesion to CSA or ICAM-1. The ratio of the quantity of cDNA of each *var* gene relative to the quantity of that gene present in a constant amount of E8B gDNA was determined by normalising with the skeleton binding protein gene.^18^ We assumed that each gene was present as a single copy in the E8B genome, and therefore, fold difference relative to gDNA allowed us to compare expression between genes as well as between samples. Where possible, we fitted linear regression curves to the data and used the equation of the resulting curves to estimate the on-switch rate of *var2csa* in ICAM-1-selected CS2 as 1.02% (0.84%, 1.35%) per generation [95% confidence interval (CI)] and the *var2csa* off-switch rate in CSA-selected E8B as 1.56% (1.35%, 1.76%) per generation (95% CI) (Fig. 6). The calculation of all gene switch rates is detailed in Methods. The *var2csa* on-switch rate in E8B was much slower than that in CS2 parasites, preventing detection of spontaneous on-switching in the absence of selection on CSA, and therefore, the E8B *var2csa* on-switch rate could not be calculated. The levels of *var2csa* cDNA in the serial samples of CSA-selected CS2 parasites fluctuated too greatly to allow accurate fitting of a linear regression curve and calculation of a switch rate.

### The mutant *var* intron is not less effective at repressing upsE

**Fig. 7:**
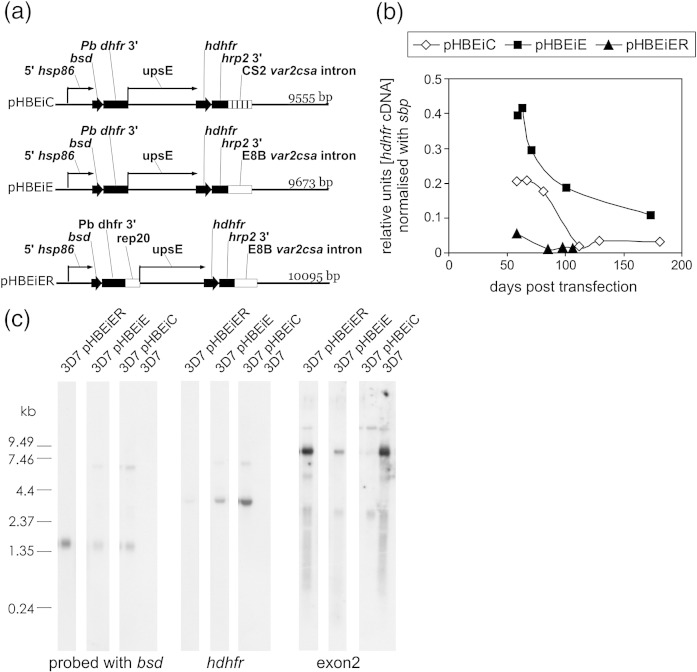
(a) Plasmids transfected into 3D7-infected erythrocytes carried the *P. falciparum* heat shock protein 86 promoter (5′ *hsp86*) driving transcription of the blasticidin deaminase gene (*bsd*) followed by the *Plasmodium berghei* dihydrofolate reductase transcriptional terminator (*Pb dhfr 3′*) followed by the ItG upsE driving transcription of human dihydrofolate reductase (hdhfr) followed by the *P. falciparum* histidine-rich protein 2 transcriptional terminator (*hrp2 3′*) followed by either the E8B or CS2 *var2csa* intron in plasmids pHBEiE and pHBEiC, respectively. The plasmid pHBEiER is the same as pHBEiE except for the inclusion of the TARE 6 (rep20) upstream of the upsE. (b) Plasmids were maintained in 3D7 parasites by growth in the presence of blasticidin-S, and the levels of *hdhfr* transcription were determined at different time points following transfection by Q-RT-PCR. Absolute quantitation was performed using standard curves of serially diluted, cloned *hdhfr* DNA of known concentration. The results were normalised using skeleton binding protein 1 cDNA levels to allow comparison of equivalent quantities of parasite material and then expressed as relative units of *hdhfr* cDNA per plasmid by dividing by the number of plasmids per genome. Plasmid number was determined by using Q-RT-PCR of transfected parasite gDNA to determine *hdhfr* normalised with *sbp*. (c) Northern blots of RNA from transfected parasites and nontransfected 3D7 parasites serially probed with sequence from *bsd*, *hdhfr*, and a conserved *var* exon 2.

It is known that the *var* intron can repress *var* genes,^12^ and thus, recombination within the CS2 *var2csa* intron was a potential cause of persistent *var2csa* activity. To determine whether the replacement of the *var2csa* intron with the chimaeric intron contributed to the persistent *var2csa* activity in CS2, we transfected 3D7 parasites with plasmids that carried either intron. These plasmids contained the upsE sequence from CS2 parasites driving transcription of *hdhfr*, followed by either the mutant *var2csa* intron from CS2 (pHBEiC) or the wild-type *var2csa* intron (pHBEiE). A third plasmid (pHBEiER) differed from pHBEiE in that it carried the TARE 6 (rep20) preceding the upsE (Fig. 7a). The plasmids also contained the blasticidin deaminase gene driven by the *P. falciparum* hsp86 promoter and were stably maintained by selection for blasticidin resistance.

The level of *hdhfr* transcripts per plasmid clearly decreased over time in parasites carrying pHBEiC, pHBEiE, and pHBEiER (Fig. 7b). Linear regression curves fitted to the first four time points of the pHBEiE and pHBEiC hdhfr cDNA levels (Fig. 7b) gave rates of decay of 1.49% per generation for pHBEiE and 1.65% per generation for pHBEiC, both of which were similar to the observed rate of 1.56% per generation for *var2csa* in E8B. The level of *hdhfr* transcribed per plasmid by parasites carrying the mutant *var* intron on pHBEiC was actually lower than the level transcribed by the wild-type intron pHBEiE parasites. Therefore, the mutant intron did not fail to silence the upsE in these constructs and we concluded that it was not responsible for the persistent *var2csa* activity in CS2 parasites.

The level of *hdhfr* transcripts in parasites carrying the rep20 construct pHBEiER was much lower than that in the other transfected parasites (Fig. 7b and c), and the *hdhfr* transcripts declined more rapidly in pHBEiER parasites than in the other transfectants to reach a low steady state (Fig. 7b). This indicated that the upsE was more effectively silenced when associated with rep20. However, Southern blots and the Sanger supercontig_0002004 sequence predicted an increase in the length of rep20 in the left-hand subtelomeric region of CS2 chromosome 12. Therefore, loss of rep20 was not responsible for decreased silencing of *var2csa* in CS2, but the observed silencing effect of rep20 on the episomal upsE indicates that subtelomeric cis sequence elements can repress *var* genes.

**Fig. 8:**
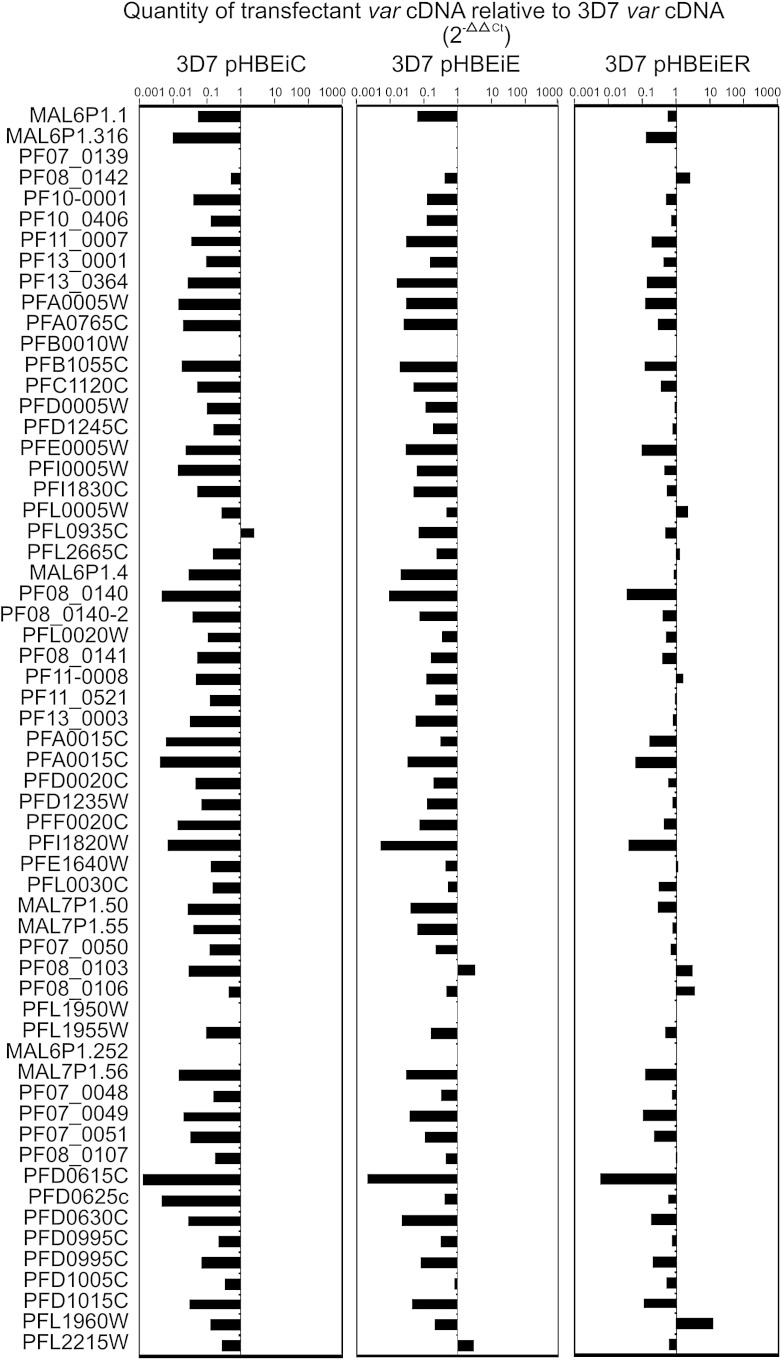
Fold differences in the cDNA levels of the transcribed *var* repertoire of transfected parasites relative to 3D7 nontransfected parasites calculated from 2^− ΔΔCt^ values normalised using arginyl tRNA synthetase.

Attempts to select transfected parasites for expression of *hdhfr* by treating with the drug WR99210 did not succeed despite detectable *hdhfr* transcripts (Fig. 7c) that, at their peak in pHBEiC and pHBEiE, were present at 24% and 23%, respectively, of the maximum level of upsE transcripts in CS2 (as determined by absolute Q-RT-PCR of *var2csa* DBL3x in CS2 using standard curves of purified plasmid containing the DBL3x sequence). Repeated independent transfections with new plasmids also generated blasticidin-resistant transfectants that transcribed *hdhfr* at similar levels but were not WR99210 resistant (data not shown). We concluded that the recently described posttranscriptional silencing exerted by an open reading frame within the upsE probably prevented translation of functional protein from the *hdhfr* transcripts.^19^ This prevented examination of parasites exclusively expressing the episomal upsE, but the observed levels of upsE activity were sufficient to down-regulate endogenous *var* gene transcription in pHBEiE and pHBEiC parasites (Figs. 7c and 8), which confirmed that the upsE activity we observed in the transfected parasites was in the biologically relevant range.

### The *var* gene introduced downstream of recombined *var2csa* is also frequently activated

The greatly diminished transcription of *hdhfr* by parasites carrying plasmids with rep20 (pHBEiER) indicated that altered subtelomeric sequences could modify gene regulation and may contribute to persistent *var2csa* expression in CS2 parasites. If differences in the subtelomeric sequences cis to *var2csa* were responsible for its persistent activity in CS2, then persistent activation might also be observed for the It4*var*44 gene that was duplicated from chromosome 7 to chromosome 12 downstream of *var2csa* in CS2. Both ICAM-1- and CSA-selected E8B parasites express It4_var44, and linear regression of It4_var44 expression over four time points in CSA-selected E8B revealed a moderate on-switch rate of approximately 0.6% per generation (Fig. 6). In both CSA- and ICAM-1-selected E8B, It4_var44 was rapidly switched off. In contrast, the ICAM-1-selected CS2 parasites maintained similar, intermediate levels of It4var44 transcripts for the 182 days examined [0.14 ± 0.01 (mean ± standard error of the mean) = 23% of the maximal levels of *var2csa* transcripts] (Figs. 5 and 6) and the CS2isC8 and CS2isD8 subclones continued to express It4_var44 at similar levels for the 270 and 184 days, respectively, they were monitored (Fig. 6). Therefore, It4_var44 is switched on rapidly in both E8B and CS2 and switched off rapidly in E8B but its activity is maintained in CS2.

## Discussion

We have provided the first evidence of ectopic recombination between *var* genes during mitosis. This mechanism and meiotic recombination of *var* genes^3^ together generate the mosaic *var* genes that contribute to the tremendous diversity of the *var* gene repertoire.^1–3^ Ectopic recombination during mitosis could increase the rate at which *P. falciparum* diversifies its repertoire of subtelomeric contingency genes and also provides parasites with the capability to generate novel variant antigens during the course of a single infection. The *var2csa* gene had recombined with an upsA gene that shared its subtelomeric location and its orientation towards the centromere, consistent with previous observations that *var* gene recombination is restricted by genomic location.^2^ The recombination initiated within the intron of *var2csa* and replaced the second exon; hence, the recombination of *var2csa* did not alter either the VAR2CSA adhesion phenotype or the VAR2CSA epitopes important for immunity.

The presence of factors required for homologous recombination and the absence of factors required for nonhomologous end joining^7^ suggests that mitotic recombination within *var2csa* in CS2 arose from double-strand break repair by non-allelic homologous recombination.^20^ Consistent with this model, there are 206 nucleotides at the start of the recombination that share 97% homology between CS2 and ItG wild-type *var2csa* and which immediately precede the divergence between CS2 and ItG *var2csa* sequences (Supplementary Fig. 1). This region of homology contains four polymorphic nucleotides between wild-type and CS2 *var2csa* that are conserved between CS2 *var2csa* and It4_var8, indicating that it corresponds to the It4_var8 sequence that was invaded by the 3′ protruding strand of the 5′ resected, broken *var2csa* duplex.^21^

The *var* gene switch rates are an intrinsic characteristic of each gene,^8^ but we have shown that intact, identical upsE promoters in the same locus in two isogenic parasites differ in their switch rate. Thus, the most probable determinants of *var* gene switch rate previously reported, the *var* gene promoter type^9^ and chromosomal location,^8,9^ do not alone account for a parasite's predisposition to express a particular *var* gene in the absence of any selective pressure. *Var2csa* is translated in CS2 parasites (Fig. 5b); thus, the increased activation of *var2csa* in CS2 is not caused by the recently described repression of PfEMP1 translation that is dependent on continual transcription of untranslated *var2csa* mRNA.^22^ Neither is it caused by loss of a gene encoding a generic *var* gene regulator because CS2 parasites can switch on and off transcription of *var* genes encoding adhesion to ICAM-1. The reversion to expression of *var2csa* by cloned ICAM-1-selected CS2 parasites showed that the increased *var2csa* on-switch rate persisted through silencing of *var2csa* and switch to expression of other *var* genes. This indicates that the altered switch rate of *var2csa* in CS2 is due to a genetic change because epigenetic memory of a *var* gene's activity is erased after silencing.^23^ Whether the *var2csa* off-switch rate is decreased in CS2 is unclear. The protracted expression of *var2csa* in CS2 could result from a normal off-switch rate paired with an on-switch rate higher than that for other *var* genes. However, there is no evidence that the continual switching predicted from such an arrangement occurred during 280 days of continuous culture of CS2 parasites as CD36 adhesion did not increase at any stage; neither were transcripts of other *var* genes detected on Northern blots probed with a conserved exon 2 probe, nor were increased levels of transcripts of any of the 17 *var* genes assayed by Q-RT-PCR detected. There are two probable explanations for the increased activation of *var2csa* in CS2. Firstly, cis sequence elements that contribute to regulation of the *var2csa* locus have been altered. Second, the parasites may have lost a trans factor that, when present, represses only *var2csa*.

The only cis sequence elements shown to regulate *var* gene transcription to date are the ups upstream sequence and the intron. The *var* intron can repress *var* promoters.^12,24^ The *var* intron also has bidirectional promoter activity^11,25^ and there are precedents in other organisms for transcription of non-coding RNAs repressing transcription of heterologous genes.^26^ The CS2 and E8B upsE were identical but recombination at the *var2csa* locus commenced within the *var2csa* intron, which led us to suspect that the chimaeric intron contributed to persistent activation of *var2csa*. We analysed the intron by transfection studies but found that the episomal CS2 *var2csa* intron was not less effective at silencing the upsE than the episomal E8B *var2csa* intron. These data strongly suggested that the mutant CS2 *var2csa* intron did not confer increased upsE activity although it is possible that the introns behave differently when integrated in the *var* gene locus.

The plasmids we used contained the hsp86 promoter that was recently shown to repress a *var* promoter.^27^ However, episomal *var* introns still repress their cognate *var* promoters by approximately 50% despite the presence of the hsp86 promoter.^28,29^ Therefore, despite the presence of the hsp86 promoter, we expected to observe greater silencing of the episomal upsE by the E8B *var2csa* intron than by the CS2 *var2csa* intron if the CS2 *var2csa* intron was responsible for the increased *var2csa* activity. In fact, we observed the reverse as the episomal upsE cis to the CS2 *var2csa* intron had less activity than the episomal upsE cis to the E8B wild-type *var2csa* intron (Fig. 7b). We concluded that any repression of the upsE exerted by the hsp86 promoter had not masked failure by the CS2 *var2csa* intron to decrease activity of the upsE. The *var* promoters that may have been silenced by hsp86 in previous studies were all silenced by default.^27–29^ In contrast, the episomal upsE was not silenced by default but retained 24% of wild-type upsE activity 60 days after transfection, indicating that the upsE was largely unaffected by the hsp86 activity. The episomal upsE was active at biologically relevant levels because it exerted considerable repression on endogenous *var* gene transcription, possibly through promoter competition for a limited pool of trans acting factors.^23^

Differences in the sequence between the telomere and *var2csa* in E8B and CS2 may be responsible for the increased activation of *var2csa* in CS2*.* This is suggested by the frequent activation in both E8B and CS2 of the It4_var44 gene that was duplicated downstream of *var2csa* in CS2. Transposition of the readily activated It4_var44 locus downstream of the *var2csa* locus may have conferred on CS2 an ability to readily activate *var2csa* similar to It4_var44, resulting in the higher on switch of *var2csa* in CS2 than in wild-type E8B. Specific sequences cis to It4_var44 and/or general structural features, such as the shorter distance from *var2csa* to the telomere in CS2, may have affected *var2csa* regulation in CS2. It4_var44 activity is maintained for far longer in CS2 than in E8B, which may be due to a faster on switch or a slower off switch in CS2 than in E8B. However, the It4_var44 locus is the same on chromosomes 7 and 12 in CS2 and chromosome 7 in E8B; thus, It4var44 probably has the same moderately high on-switch rate in both CS2 and E8B, leading to frequent activation in both parasites. Because there are two copies of It4_var44 in CS2, their cumulative activity could maintain a more constant, intermediate level of It4_var44 transcript abundance in CS2 than in E8B.

The greatly reduced activity of the upsE on the plasmid carrying the rep20 proved that subtelomeric, cis sequence elements can affect upsE activity, although lack of rep20 itself was probably not responsible for the diminished silencing of *var2csa* in CS2. In contrast, rep20 does not affect upsB and upsC activity.^28,29^ Rep20 presumably constrained the upsE plasmid mobility by strong, random association with primarily silent telomeric clusters at the nuclear periphery.^30^ The upsB and upsC sequences must themselves form associations with silent telomeric clusters that are sufficiently strong to render the association mediated by rep20 redundant. Interestingly, the CS2 *var2csa* gene does not leave telomeric clusters to be transcribed,^31^ whereas the *var2csa* genes in 3D7 parasites and FCR3 parasites (which are isogenic with CS2) do leave telomeric clusters when transcribed.^22,32^ Subtelomeric sequences determine telomere localisation to telomeric clusters.^14^ Hence, differences between CS2 parasites and FCR3 and 3D7 parasites in the subtelomeric sequences contiguous with *var2csa* may contribute to the different mobilities of the *var2csa* locus in these parasites.

Subtelomeric sequences cis to the *var2csa* locus could determine the rate of *var2csa* activation by binding trans factors required for epigenetic activation or silencing. Both trans factors *Sir2* and *Orc1* bind subtelomeric sequences, and *Sir2* binding spreads upstream and silences *var2csa*.^4,33,34^ Doubtless, other sequences cis to *var2csa* are also required for typical regulation of *var2csa*, including an unknown cis sequence that recruits the trans factors responsible for trimethylation of lysine 9 of histone 3 in silent *var2csa*.^6^ Sequences cis to *var2csa* could also bind trans factors required for formation of chromatin loops that are tethered in transcriptionally active or repressed subnuclear domains.^35^

Effective *var* gene switching is obviously important for evasion of the immune response but the biological significance of the different *var* gene switch rates is unclear. PfEMP1s expressed by parasites causing severe malaria appear to be more commonly encountered than PfEMP1s expressed by parasites causing non-severe malaria, but this frequency decreases with increasing host age and presumably with acquisition of effective immunity.^36,37^ Conversely, expression of the upsA and upsB *var* genes is associated with more severe disease, but the upsC *var* genes are expressed more frequently in the absence of selective pressure.^9,38,39^ It has been proposed that their adhesion phenotype confers a selective advantage on the more pathogenic and less frequently expressed upsA and upsB *var* genes, but development of host immunity removes this advantage, allowing the more frequently expressed and less pathogenic upsC *var* genes to predominate.^9^ Thus, the difference in switch rate between different *var* genes may be unimportant for the prevalence of severe disease in children but will contribute to the observed pattern of infection in older individuals and may protect the parasites antigenic repertoire.

We have shown that the ups type, the *var* intron, and chromosomal location are insufficient to determine the switch rate. We have also shown that the cis sequence element rep20 can affect *var* transcription and our findings indicate that other cis sequence elements lying outside subtelomeric *var* genes contribute to their intrinsic switch rate. Further investigation of the subtelomeric sequences of CS2 parasites will provide valuable insights into the mechanisms *P. falciparum* employs to initiate *var* gene switching, a critical component of the process of antigenic variation.

## Methods

### Plasmodium culture, selection for adhesion phenotype, assay of adhesion phenotype, and transfection

The Brazilian *P. falciparum* isolate IT was cloned twice by Dr. L. H. Miller (National Institutes of Health) to generate the clone ItG2F6. In our laboratory, ItG2F6 was cloned again to generate the clone FAF6.^40^ A line derived in our laboratory from FAF6 for adhesion to endothelial cells was named interchangeably FAF-EA8^41^ or E8B.^42^ E8B was selected for adhesion to CSA to generate CS2.^43^ We cloned CS2 prior to this study. Therefore, E8B was derived from the IT isolate by three serial clonings by limiting dilution and CS2 was derived from E8B by one limiting dilution cloning. The IT parasites are isogenic with parasites frequently described as FCR3, presumably due to an early contamination.^44,45^

*P. falciparum* parasites were maintained in culture as previously described.^46^ The parasite culture medium was supplemented with either 0.5% albumax II (Gibco) for nontransfected parasites or 5% heat-inactivated human serum 0.25% albumax for transfected parasites. CS2 and E8B parasite lines were selected for high-level adhesion to receptors immobilized on plastic Petri dishes by repeated panning.^46^ The receptors bound to the dishes were 50 μg/ml bovine trachea CSA (BT-CSA) (Sigma), 8 μg/ml recombinant ICAM-1 (Bender MedSystems), and 15 μg/ml recombinant CD36 (R&D Systems). 3D7 parasites were transfected with 80 μg of purified plasmid (Qiagen)^47^ and selected for resistance to 2 μg ml^− 1^ blasticidin-S 4–6 h after transfection.

### Cloning

The wild-type It4_var4 (ItG *var2csa*) sequence from within the first exon to within the second exon was amplified from E8B gDNA and cloned and sequenced. The mutant CS2 *var2csa* sequence from the end of the first exon to the end of the second exon was amplified using the forward primer GGTATTGCGTTGGCGTTAGG and the degenerate second exon reverse primer ATATCCAHTTCTTCATAYTCACTTTC. The second exon sequence was used to prime amplification of 2645 nucleotides of unknown sequence downstream of CS2 *var2csa* by Universal Fast Walking PCR^13^ using the oligonucleotides primer 1, ATACATCCCCAAACCTACG; primer 2, TATCCCTACACGTCACCTAANNNNNNNNNN; primer 3, AGTGATAGTGGACACTACTACGAA; and primer 4, CAAGTGGAAATAGTTCAACAAATACC. The *var2csa* upstream sequence (upsE) was amplified from both CS2 and E8B parasites as 2256 nucleotides of sequence 5′ of the *var2csa* coding sequence. PCR products were cloned and sequenced by standard methods.

The plasmid pHBCAM^R^ ^28^ was digested with EcoRI and ligated to either CS2 or E8B *var2csa* intron sequences that were engineered with complementary MfeI sites at both ends. These plasmids were digested with PstI and BamHI and then ligated with the 2256-bp CS2 *var2csa* upstream sequence (upsE) that had been amplified with complementary PstI and BamHI sites. The resulting plasmid carrying the E8B wild-type *var2csa* intron was named pHBEiE, and the plasmid carrying the CS2 mutant *var2csa* intron was named pHBEiC. The plasmid pHBEiER was made by ligating PstI-digested pHBEiE with a 540-nucleotide rep20 sequence with complementary PstI sites at each end.

### Southern blots

Separation of *P. falciparum* chromosomes by PFGE was performed as previously described.^48^ PFGE of digested gDNA was performed on the BioRad CHEF-DR II apparatus using a modification of the manufacturer's method; briefly, electrophoresis through 1% agarose in 0.5 × TBE was performed for 14 h at 6 V/cm using a ramped switch time from 1 to 6 s. Southern blots of conventional gels and PFGE were probed with [α-^32^P]dATP random prime-labelled DNA. Southern blots of digested gDNA were probed with sequence from nucleotides 7354 to 7607 at the 3′ end of the CS2 *var2csa* first exon. Southern blots of PFGE-separated chromosomes were probed with sequences from the 3′ end of It4_var44 (nucleotides 5557–5993) and the middle of ItG *var2csa* (It4_var4) (nucleotides 2275–3406). Southern blots were washed with 0.2 × SSC 0.1% sodium dodecyl sulfate (SDS) at 65 °C.

### RNA extraction and Northern blots

Trizol reagent (Sigma)^49^ was used to extract RNA from early ring stage parasite cultures at approximately 8 h post-invasion. Northern blots were performed using 0.9% agarose gels and probed with [α-^32^P]dATP random prime-labelled DNA as previously described.^50^ The ItG *var* gene second exon probe was described previously.^51^ The *var2csa* and It4_var44 exon 1 probes were the same as those used to probe PFGE-separated chromosomes. Northern blots probed with exon 2 sequences were washed with 2 × SSC and 0.1% SDS at 55 °C. Northern blots probed with specific exon 1 sequences were washed with 0.5 × SSC and 0.1% SDS at 60 °C.

### Quantitative RT-PCR

All Q-RT-PCR was performed using SYBR PCR master mix (Applied Biosystems) on a PE7900HT (Applied Biosystems).

Genes identified as possibly deleted or duplicated by microarray were quantitated in both CS2 and E8B gDNA using specific primers (Supplementary Table 2). A single gDNA serially diluted 5 times over 5 logs of concentration was used to create standard curves for each set of primers from which the levels of each gene in the CS2 and E8B gDNA samples were interpolated from their Ct values. These levels were normalised by dividing by the levels of the skeleton binding protein gene *sbp* determined by the same approach in each sample. The resulting relative values compare the level of each gene in equivalent amounts of CS2 and E8B gDNA.

Q-RT-PCR of 17 ItG *var* genes was performed to determine switch rates. Each primer pair was used to amplify a 5-point, 5-log dilution series of CS2 gDNA, and the gradient of the curve of threshold cycle *versus* concentration was used in the equation 10^[− 1/gradient]^ to calculate the efficiency (*E*) of each PCR reaction.^52^ This value for each gene was then used to calculate the ratio of its expression in each cDNA relative to the number of copies of the gene present in a constant amount of E8B gDNA using the skeleton binding protein 1 gene (SBP) (PFE0065w) to normalise in the equation ratio = ((*E*_*var* gene_)^ΔCP*var* gene(E8B gDNA–cDNA sample)^)/((*E*_SBP_)^ΔCP_SBP_(E8B gDNA–cDNA sample)^),^52^ where crossing point (CP) was equivalent to the threshold cycle (Ct) determined using SDS software (Applied Biosystems). The primer sequences and PCR efficiencies are provided in Supplementary Table 2.

Absolute quantitation of *hdhfr* and *bsd* in transfectant cDNA and gDNA by Q-RT-PCR was performed using the primers listed in Supplementary Table 2. Standard curves were made using purified plasmids of known concentration containing the target gene sequences as previously described.^50^ The relative quantities in transfectant cDNA and gDNA of the skeleton binding protein gene (*sbp*) were determined by Q-RT-PCR using the primers listed in Supplementary Table 2 to amplify standard curves of diluted 3D7 isolate gDNA.^18^ Relative *sbp* levels were used to normalise the *bsd* and *hdhfr* levels, and the normalised cDNA data were divided by the normalised gDNA data to calculate relative levels of *hdhfr* and *bsd* cDNA per plasmid.

Relative quantitation of the 3D7 *var* repertoire by 2^− ΔΔCt^ analysis was performed using a previously described primer set^53^ supplemented with previously described additional primers.^9,54,55^ Analysis was performed using arginyl tRNA synthetase to normalise^54^ and wild-type 3D7 cDNA as a calibrator. The following genes were amplified by the same set of primers and are indicated elsewhere by the first gene accession only: PFC1120c/PFC0005w, PFA0015c/PFI1820w/MAL6P1.314, PFD1235w/MAL7P1.1, PFL1955w/PFL1970w, PFD0630c/PFD0635c, and PFD0995c/PFD1000c.

### Gene switch rates

Switch rates of *var2csa* were determined by fitting linear regression curves to *var2csa* cDNA levels. Maximum *var2csa* transcript levels were determined as 0.62 relative to gDNA from the average of the maximum values for the CSA-selected CS2 and E8B time courses (Fig. 6). Inspection of the ICAM-selected CS2 parasites *var2csa* regression curve revealed a linear increase throughout the entire time period with 0 *var2csa* cDNA predicted for day 1 by the regression equation *y* = (0.003185 ± 0.0001581)*x* + (−0.004249 ± 0.1727) (± 95% CI) (*r*^2^ = 0.9927). Therefore, the on-switch rate/generation was determined by dividing 100 by the solution of the equation for *x* (number of days) divided by 2 when the maximum level of *var2csa* cDNA was substituted for the *y* value. The levels of *var2csa* in the CSA-selected E8B parasites did not decline in a uniformly linear fashion but was preceded by a plateau of maximal transcript levels followed by a rapid decline and then a much slower decline from low levels to silent. Linear regression was fitted to the four time points in the middle of the curve corresponding to the maximal rate of decline (Fig. 6) to generate the equation *y* = (− 0.004829 ± 0.0006827)*x* + (0.8391 ± 0.07639) (± 95% CI) (*r*^2^ = 0.9616). The off-switch rate was determined by dividing 100 by the difference of the solutions of the equation for *x* (number of days) at *y* = 0 and *y* = maximum *var2csa* [cDNA] and then dividing the result by 2. This approach assumed that no net switching away from *var2csa* had occurred for the first 47 days of culture during which *var2csa* cDNA levels remained maximal.

E8B grown after selection on CSA also transiently expressed It4_var44 at sufficient levels to allow linear regression over four time points; the slope of the resulting equation, *y* = (0.001736 ± 0.000353)*x* + (− 0.08402 ± 0.0395) (± 95% CI) (*r*^2^ = 0.9236) was 0.55 times that of *var2csa* in CS2 selected on ICAM-1, indicating an on-switch rate for It4_var44 of approximately 0.55 × 1.02 = 0.6% per generation in E8B.

Linear regression curves were fitted to the first four time points of the pHBEiE and pHBEiC *hdhfr* cDNA levels presented in Fig. 7b. The resulting equations for pHBEiE, *y* = (− 0.005285 ± 0.001191)*x* + (0.7115 ± 0.08967) (± 95% CI) (*r*^2^ = 0.9079), and for pHBEiC, *y* = (− 0.003643 ± 0.0007271)*x* + (0.4415 ± 0.05969) (± 95% CI) (*r*^2^ = 0.9262), were used to calculate the percentage of episomal upsE silencing per generation as (100/(*x* intercept/2)) to give values of 1.49% per generation for pHBEiE and 1.65% per generation for pHBEiC.

### Western blot

Triton-X-100-insoluble, SDS-soluble fractions of parasite cultures were extracted; separated by SDS-PAGE; transferred to nitrocellulose membrane; and probed with polyclonal rabbit antiserum raised to the conserved acidic terminal sequence of PfEMP1 as previously described.^56^

### DNA microarray hybridisation

gDNA (12 μg) from CS2, E8B, and P1B5 (IT sequence strain†) parasites were fragmented by a frequent cutter restriction enzyme ApoI for 2 h at 50 °C and then heat inactivated for 20 min at 80 °C. After phenol-chloroform extraction and isopropanol precipitation, the fragmented DNAs were resuspended in water and end labelled with 25 nmol of Biotin-N11-ddATP (NEN) using a terminal transferase (Roche) following the manufacturer's instructions. Biotin-labelled gDNA were then added to the Affymetrix hybridisation cocktail (standard eukaryotic Affymetrix protocol) and hybridised to the PFSANGER arrays overnight at 65 °C in a rotating oven (60 rpm).

The high-density 8-μm PFSANGER tiling-like array has been described elsewhere.^57^ Following hybridisations, the chips were washed, stained, and scanned according to the manufacturer's recommendations. P1B5 hybridisation was not performed the same day as E8B and CS2, but the three raw files were normalised together for analysis. The hybridisation intensity for each 25-mer feature was computed using Affymetrix GCOS v1.3 software, and the CEL files were transferred into the R/Bioconductor environment.^58^ For DNA–DNA analysis, the chips were RMA preprocessed^59^ using the affy package^60^ and a modified chip definition file generated in-house (using the altcdfenv package) containing 5677 probesets = 5358 genes (*P. falciparum* genome genedb-release 2005‡). Log2 ratio of CS2/E8B, CS2/P1B5, and E8B/P1B5 were calculated for each gene and compared to assess gene amplification and/or deletion [over log2 (1) or under log2 (− 1), respectively].

## Appendix A

Supplementary Fig. 2 Alignment of intron sequences from the recombined CS2 *var2csa*, the donor It4_var8, and the recipient wild-type E8B *var2csa* (It4_var4). “Start” above the alignment indicates the start of the homologous sequence predicted to precede the *var2csa* breakpoint. Asterisks below the alignment indicate nucleotide polymorphisms conserved between CS2 *var2csa* and It4_var8 that lie within the region of homology predicted to correspond to the protruding 3′ invading strand of *var2csa.* The underlined sequence is *var* intron region 2. Blocks in black background are the conserved repeat sequences that are inverted in *var* intron region 3 relative to region 1.

## Footnotes

† www.sanger.ac.uk

‡ www.genedb.org
