# Supplementary material for: Ectopic Recombination of a Malaria var Gene during Mitosis Associated with an Altered var Switch Rate
Source: J Mol Biol. 2009 Jun 12;389(3):453–69. doi: 10.1016/j.jmb.2009.04.032 (PMC3898907; doi:10.1016/j.jmb.2009.04.032)
Supplement: Supplementary Fig. 2 — Alignment of intron sequences from the recombined CS2 var2csa, the donor It4_var8, and the recipient wild-type E8B var2csa (It4_var4). “Start” above the alignment indicates the start of the homologous sequence predicted to precede the var2csa breakpoint. Asterisks below the alignment indicate nucleotide polymorphisms conserved between CS2 var2csa and It4_var8 that lie within the region of homology predicted to correspond to the protruding 3′ invading strand of var2csa. The underlined sequence is var intron region 2. Blocks in black background are the conserved repeat sequences that are inverted in var intron region 3 relative to region 1. [file mmc1.doc]

Suppl fig 2

10 20 30 40 50 60

....|....|....|....|....|....|....|....|....|....|....|....|

**It4_var8** 1 **GTAATATATATATATATATATATATATAT ..ATAT........................**

**CS2**  1 **GTAATATATATATATATATATATATGTGTTGTATATGTATGTATGTATGTATGTATGTAT**

**It4_var4** 1 **GTAATATATATATATATATAT..GTGTGTTGTATATGTATGTATGTATGTATGTATGTAT**

70 80 90 **start** 110 120

....|....|....|....|....|....|....|....|....|....|....|....|

**It4_var8** 34 **A....TATATATGTAT..ATGTA.GTGTT....TATGTATGTATGTATGTATGTTATGTG**

**CS2**  61 **GGA.GTATGTATGTATGTATGTATGTATGTATGTA.GTATGTATGTATGTATGTTATGTG**

**It4_var4** 59 **GTATGTATGTATGTATGTATGTATGTATGTATGTATGTATGCATGTATGTATGTTATGTG**

*

130 140 150 160 170 180

....|....|....|....|....|....|....|....|....|....|....|....|

**It4_var8** 83 **TATATGTTATATATGTATTTAATATATGTATTTATATTGGAAAAGAAAAAAGGAAAAAGT**

**CS2**  119 **TATATGTTCTTTATGTATTTAATATATGTATTTATATTGGAAAAGAAAAAAGGAAAAAGT**

**It4_var4** 119 **TATATGTTATATATGTATTTAAAATATGTATTTATATTGAAAAAGAAAAAAGGAAAAAGT**

* *

190 200 210 220 230 240

....|....|....|....|....|....|....|....|....|....|....|....|

**It4_var8** 143 **AATATAGGAATATATCTATTAAAAAAAAAAAGAGAGAGATTTTAGAATAATAAAAATAGA**

**CS2**  179 **AATATAGGAATATATCTATTAAAAAAAAAA.GAGAGAGATTTTAGAATAATAAAAATAGA**

**It4_var4** 179 **AATATAGGAATATATCTATTAAAAAAAAAAAGAGAGTGATTTTAGAATAATAAAAATAGA**

*

250 260 270 280 290 300

....|....|....|....|....|....|....|....|....|....|....|....|

**It4_var8** 203 **AAAAATGAAAATAAAAATTTTTATAAAATAAAAAATGAGAAATATCATCAAAGAAAAAAA**

**CS2**  238 **AAAAATGAAAATAAAAATTTTTATAAAATAAAAAATGAGAAATATCATCAAAGAAAAAAA**

**It4_var4** 239 **AAAAATGAAAATAAAAATTTTTATAAAATAAAAAATGAGAAATATCATCAAAGAAAAAAA**

310 320 330 340 350 360

....|....|....|....|....|....|....|....|....|....|....|....|

**It4_var8** 263 **AA.........AAAAA.TTCATTAAA....AAAATATGTAAAAATAAAA..AAAATATAA**

**CS2**  298 **AA.........AAAAAATTCATTAAA....AAAATATGTAAAAATAAAA..AAAATATAA**

**It4_var4** 299 **AACAATTTATTAAAAAACTAATTAAACTTAAAAATAAATAAAAAAAAAATTAACATATAA**

370 380 390 400 410 420

....|....|....|....|....|....|....|....|....|....|....|....|

**It4_var8** 307 **AAAAGAAAATTATTAGAAA...AAATTAATTACAAATAAAATAAAAAAAAAATTTATTAA**

**CS2**  343 **AAAAGAAAATTATTAGAAA...AAATTAATTACAAATAAAATAAAAAAAAAATTTATTAA**

**It4_var4** 359 **ATTTTTATAAAATAAGAAATGGAAATTTTATAAAAAAAAAAAAAAAAAAAATTTCATTAA**

430 440 450 460 470 480

....|....|....|....|....|....|....|....|....|....|....|....|

**It4_var8** 364 **AAATAAAATAAAAAAAAA.ATTTATTAAAAA...TAAAACAA.......AAAAAAAAATT**

**CS2**  400 **AAATAAAATAAAAAAAAA.ATTTATTAAAAA...TAAAACAA.......AAAAAAAA.TT**

**It4_var4** 419 **ATAAAAAATAAAAAAAAATATTTAACAAAAAAATTAAAAAAATTTATTTACAAAAAAAAT**

490 500 510 520 530 540

....|....|....|....|....|....|....|....|....|....|....|....|

**It4_var8** 413 **TATTAAAAATAAA..ACAAAAAAAA.....AAAAATTT......ATTAGAAATAAA....**

**CS2**  448 **TATTAAAAATAAA..ACAAAAAAAA.....AAAAATTT......ATTAGAAATAAA....**

**It4_var4** 479 **TATAAAAATTTATTTACAAAAAAAATTTAAAAAAATTTTATACAATGAGAATTAAATGGA**

550 560 570 580 590 600

....|....|....|....|....|....|....|....|....|....|....|....|

**It4_var8** 455 **.......ATAAAATAAAATAAAAATAAAATTTATTACAAATAAAAAATG...ATT..AAA**

**CS2**  490 **.......ATAAAATAAAATAAAAATAAAATTTATTACAAATAAAAAATG...ATT..AAA**

**It4_var4** 539 **AATTTAAAAAAAAAAAAATAAAGAAAAAATATTTATAAAATGAGAAATTGAAATTTAAAA**

610 620 630 640 650 660

....|....|....|....|....|....|....|....|....|....|....|....|

**It4_var8** 504 **AAAAAATTTATTAAATAAAAAAAA.........AAAAAAAAAAAAAAAAAATACAT..AA**

**CS2**  539 **AAAAAATTTATTAAATAAAAAAAA.........AAAAAAAAAAAAAAAAA.TACAT..AA**

**It4_var4** 599 **AAAAAAAAAAGTATAAAAAAAAAATTATGTTTAAAAAAAAAAAAAAAAAATTACATGCAT**

670 680 690 700 710 720

....|....|....|....|....|....|....|....|....|....|....|....|

**It4_var8** 553 **ATATATGCACA.....TATATATTTATACATATATATATACCCATACATACATATATATG**

**CS2**  587 **ATATATGCACA.....TATATATTTATACATATATATATACCCATACATACATATATATG**

**It4_var4** 659 **ATATATGCACAAAATTTATTTATATATACATACATACATACCCATACAAATATATATATA**

730 740 750 760 770 780

....|....|....|....|....|....|....|....|....|....|....|....|

**It4_var8** 608 **CA..TACCCATACATACATATACATGTA..TACCCATACATAAATATACATGTATACCCA**

**CS2**  642 **CA..TACCCATACATACATATACATGTA..TACCCATACATAAATATACATGTATACCCA**

**It4_var4** 719 **CACATACCCATACAAATATATATATATACATACCCATACATACATATATATATATACCC.**

790 800 810 820 830 840

....|....|....|....|....|....|....|....|....|....|....|....|

**It4_var8** 664 **TACATACATATATATAAATACCTAAAAATACATATATATATACATCCCCAAACCTACGTA**

**CS2**  698 **TACATACATATATATAAATACCTAAAAATACATATATATATACATCCCCAAACCTACGTA**

**It4_var4** 777 **..CA.ACATATACATTCACATGCATACATATCTACCTAAATACATACACATATAAATACT**

850 860 870

....|....|....|....|....|....|....|

**It4_var8** 724 **TACATACACATATACGTGTGTATCCGTTTTTATAG**

**CS2**  758 **TACATACACATATACGTGTGTATCCGTTTTTATAG**

**It4_var4** 835 **TACATACATATATATAT.TCAATTTTTTATTCTAG**
