# Supplementary material for: Ectopic Recombination of a Malaria var Gene during Mitosis Associated with an Altered var Switch Rate
Source: J Mol Biol. 2009 Jun 12;389(3):453–69. doi: 10.1016/j.jmb.2009.04.032 (PMC3898907; doi:10.1016/j.jmb.2009.04.032)
Supplement: Supplementary Table 2 [file mmc3.doc]

Supplementary Table 2

Q-RT-PCR oligonucleotides and gradients of Ct plotted against gDNA concentration for a 5 log dilution series of CS2 gDNA amplified for each set of primers

| accession | gene | adhesion phenotype | domain for  Q-RT-PCR | Gradient of Ct vs concentration for 5 logs of a CS2 gDNA dilution series | Q-RT-PCR  forward oligonucleotide  reverse oligonucleotide |
| --- | --- | --- | --- | --- | --- |
| AJ133811 | *FCR3.varCSA* | CSA | dblα | -3.24218 | GACGCGAAACGAAACCGTAA  ACTACTTGGGCCACAATTTTTTG |
| AF275870/ EF158103 | *var* p/ It4_var6 |  | dblα | -3.3164 | GAAGAATGTGGACAAAGCAAAAGA  TTATTGTCGGCATCACCTTTGTAG |
| AF275856 | *var* y |  | dblα | -3.25378 | TGGTGATGCACTAAATCCATCTCT  CTCACTTTTGCCGGGCTTT |
| AF193424 | *A4tres var* | CD36 ICAM-1 | dblα | -3.33177 | ATAATATGCACAGCACCTGGTACTG  GGAGGCTCTCCATTGACACATC |
| AF275849/  EF158075 | *var* i/ It4_var19 |  | dblα | -3.27496 | TGTGGTTCAAGAGAGAAGGGAAGT  CAATCACACATCGGCATTTACCT |
| AF134154 | *var-CS2* | CSA | dblγ | -3.26216 | AATGGTCTCAGTGAAAGCGAGAA  CAGTTTGTTGTATGAGTAATCCTGCAAT |
| AF275862/ EF158085 | *var*E/ It4_var33 |  | dblα | -3.3383 | ATGGAGGATTTGACAAATGATCAG  AAAAAAATTATCACCGTCACCATTG |
| AF275854/ EF158076 | *var* w/ IT4_var22 |  | dblα | -3.1317 | CCAACAGGTGCTGATTATTTTAGAAA  TTGTAAAAACATTAGTACCATCTGAACCTTT |
| L42244 | *A4var* | CD36 | dblγ | -3.48741 | GTGGTAGTCAAGAGAATGGTAAGAAAGA  TTTGCAGTTTGTTGTTCATATTGATC |
| AJ007941/ EF158098 | *var*^CD36^ /It4_var5 | CD36 | dblα | -3.34616 | GACCAACATGTGGAGGTAATGAAA  TGGTCGGTCTCTTTTGTGAATTTT |
| AJ007942 | *var* aa/*var*^ICAM-1^ | ICAM-1 | dblα | -3.3199 | TATAAAGAATTGACGGCGACGA  GCGATCTCTTAGCGCCACAT |
| AY349173 | *var*EHA |  | 3' exon 1 | -3.25776 | AACACTATAAATTTGATGACACAGAGATGA  GGTTCTATAGCTTTGTTAACTTCACTTTCA |
| AF003473 | FCR3S1.2-*var1* | rosetting, CD36 | dblα | -3.36797 | ACATGCAGCAAGGAGCTTGA  TTTGTGATGGACCTTGTCCAGTA |
|  |  | PECAM-1/CD31 |  |  |  |
|  |  | blood group A antigen |  |  |  |
|  |  | non-immune IgM |  |  |  |
|  |  | heparan sulfate like GAG |  |  |  |
| AF275866/ EF158084 | *var*L/ It4_var30 |  | dblα | -3.06185 | GGAGGTGATGGAAAACATTCAAC  CGGTGTCATTTTTGCCCTTT |
| AF275867 | *var*M |  | dblα | -3.31982 | GTGACGAAGGGAGGGAAGAAT  CACTTCCGTAGCGTGTTTTTAGAG |
| AF275864 | It4_var44 |  | 3’ exon 1 | -3.50944 | TGCTTCATATTTTCGACCAACGT  CAGCGGCATCGGTTTTG |
| AY372123 | IT4 var4 | CSA | dbl3x | -3.40556 | TGTCATGCTGTTCAAAGAAGTTTTATT  TGGTACCCAAAATCATATTCTTATAATCA |
|  | *hdhfr* |  |  |  | TGAATCACCCAGGCCATCTT  AAGTCTTGCATGATCCTTGTCACA |
|  | *bsd* |  |  |  | CTCAAGAAGAATCCACCCTCA  TGCTGTTGATTGTAGCCGTT |
| PFE0065w | *sbp* |  |  | -3.26431 | TTAGCCGACGAACCAACACA  TTCGGTTGTCTCTGGTACTGCA |
| PF10_0304 |  |  |  |  | AAACATTATATCATGCAATGATTCATATTG  TTTCGTCATCGGAGTTTTTATCAA |
| PF11_0472 |  |  |  |  | AAGGAAAAAGAGCTAGTTATAATAAATTTGGT  AAAGTATAACTGAATCATCATTAACTGTTCAA |
| PF14_0762 |  |  |  |  | ACACCTTTAGGGACAATAAAGGATATAATT  TTCAACTTATCCTTGATTATTTCATCTTTT |
| pf10_0253 |  |  |  |  | CGAATAAGAGATTCACCGGTTGA  TTTCCGAGCCCTCATGATATTAG |
| PF10_0304 |  |  |  |  | AAACATTATATCATGCAATGATTCATATTG  TTTCGTCATCGGAGTTTTTATCAA |
|  |  |  |  |  |  |
